# Investigation of factors affecting the stability of compounds formed by isovalent substitution in layered oxychalcogenides, leading to identification of Ba_3_Sc_2_O_5_Cu_2_Se_2_, Ba_3_Y_2_O_5_Cu_2_S_2_, Ba_3_Sc_2_O_5_Ag_2_Se_2_ and Ba_3_In_2_O_5_Ag_2_Se_2_†

**DOI:** 10.1039/d1tc05051f

**Published:** 2022-02-08

**Authors:** Gregory J. Limburn, Daniel W. Davies, Neil Langridge, Zahida Malik, Benjamin A. D. Williamson, David O. Scanlon, Geoffrey Hyett

**Affiliations:** School of Chemistry, University of Southampton Southampton SO17 1BJ UK g.hyett@soton.ac.uk; Department of Chemistry, University College London, 20 Gordon Street London WC1H 0AJ UK; Research Computing Service, Information and Communication Technology, Imperial College London London SW7 2AZ UK; Department of Materials Science and Engineering, Norwegian University of Science and Technology (NTNU) Trondheim 7491 Norway

## Abstract

Four novel compositions containing chalcogenide layers, adopting the Ba_3_M_2_O_5_M′_2_Ch_2_ layered structure have been identified: Ba_3_Sc_2_O_5_Cu_2_Se_2_, Ba_3_Y_2_O_5_Cu_2_S_2_, Ba_3_Sc_2_O_5_Ag_2_Se_2_ and Ba_3_In_2_O_5_Ag_2_Se_2_. A comprehensive comparison of experimental and computational results providing the crystallographic and electronic structure of the compounds under investigation has been conducted. Materials were synthesised at 800 °C under vacuum using a conventional ceramic synthesis route with combination of binary oxide and chalcogenide precursors. We report their structures determined by Rietveld refinement of X-ray powder diffraction patterns, and band gaps determined from optical measurements, which range from 1.44 eV to 3.04 eV. Through computational modelling we can also present detailed band structures and propose that, based on their predicted transport properties, Ba_3_Sc_2_O_5_Ag_2_Se_2_ has potential as a visible light photocatalyst and Ba_3_Sc_2_O_5_Cu_2_Se_2_ is of interest as a p-type transparent conductor. These four novel compounds are part of a larger series of sixteen compounds adopting the Ba_3_M_2_O_5_M′_2_Ch_2_ structure that we have considered, of which approximately half are stable and can be synthesized. Analysis of the compounds that cannot be synthesized from this group allows us to identify why compounds containing either M = La, or silver sulfide chalcogenide layers, cannot be formed in this structure type.

## Abstract 2

In this paper we identify a novel p-type conductor and a photocatalyst both adopting layered oxychalcogenide structure types, and also identify the factors which affect the stability of such compounds.

## Graphical abstract

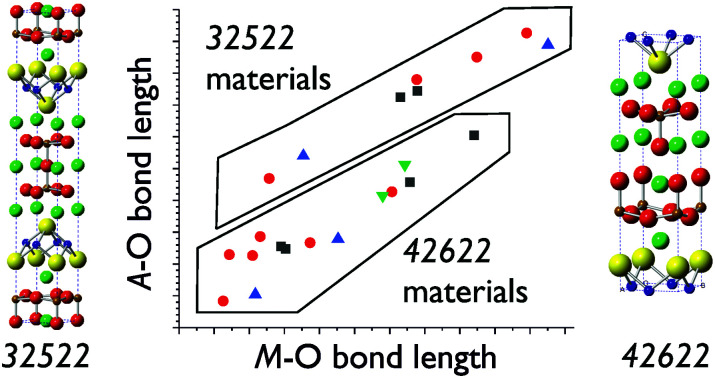

## Introduction

The mixed-anion layered material Sr_3_Sc_2_O_5_Cu_2_S_2_ has been identified as a promising p-type transparent conductor, having an unusually high hole mobility,^1,2^ which subsequently prompted a computational search for further p-type conductors adopting this structure.^3^ Isostructural oxysulfides, Ba_3_Sc_2_O_5_Cu_2_S_2_ and Ba_3_In_2_O_5_Cu_2_S_2_, have been found to be photocatalysts,^4^ in common with other layered oxysulfides.^5,6^ The 32522 structure of Sr_3_Sc_2_O_5_Cu_2_S_2_ – the designation derived from the ratio of the ions – adopted by these compounds is part of a broader class of oxychalcogenides with isostructural copper or silver chalcogenide anti-litharge layers, separated by oxide layers with varying thickness and co-ordination, often adopting a fragment of the perovskite structure.^7–9^ The separation of the ‘heavy’ chalcogenide layers by the ‘light’ oxide layers in these materials creates a quantum well leading to hole transport in the chalcogenide layers combined with a large band gap,^10,11^ resulting in the photocatalysis and p-type conducting functions observed in these materials.

**Fig. 1:**
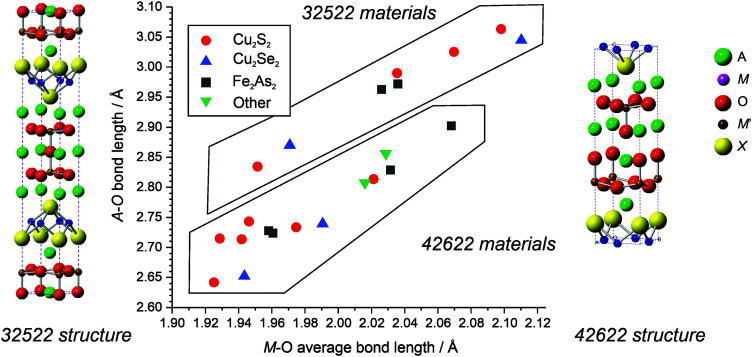
Structure map of layered mixed anion phases showing adoption of either 32522 or 42622. Examples with copper sulphide heavy layers are indicated by red circles, copper selenide by blue triangles, iron arsenides by black squares, and the less common layers (iron phosphide, cobalt arsenide, nickel phosphide and nickel arsenide) by green triangles.

Specifically, in the 32522 structure, A_3_M_2_O_5_M′_2_X_2_, the ‘heavy’ anion layer [M′_2_X_2_]^2−^ adopts the anti-litharge structure, while the oxide layer [A_3_M_2_O_5_]^2+^ adopts a triple layer fragment of the perovskite structure, but truncated such that the geometry of the M ion is pyramidal rather than octahedral. There is a competing and related structure type which can be formed using ions with the same formal charges, the 42622, with general formula A_4_M_2_O_6_M′_2_X_2_, sometimes expressed as the empirical formula AMO_3_M′X. In comparison to the 32522 structure, the 42622 has an additional rock-salt structured AO layer which shears and displaces the vertex sharing of the apical oxygen of the MO_5_ polyhedra. Schematic unit cells for the two structures are shown in Fig. 1. It has been previously identified that there is a delicate balance between the stability of the 42622 or 32522 structure for a given combination of ions.^12^ For example, Sr_4_Ga_2_O_6_Cu_2_S_2_ is stable, but the 32522 equivalent is not. Replacing gallium with scandium, it is found that both structures, Sr_4_Sc_2_O_6_Cu_2_S_2_ and Sr_3_Sc_2_O_5_Cu_2_S_2_, can be formed. With further substitution of strontium by barium it is found that only the 32522 structure is formed, Ba_3_Sc_2_O_5_Cu_2_S_2_. The overall trend is that moving from smaller ions (Sr^2+^, Ga^3+^) to larger ions (Ba^2+^, Sc^3+^) leads to a preference for the 32522 structure over the 42622 structure.

A survey of the literature indicates that there are a total of 10 compounds adopting the 32522 structure, where full structural details are available. The 42622 is slightly more numerous with 14 examples known. These mixed anion compounds have been found with a range of trivalent M ions in the [A_4_M_2_O_6_]^2+^ or [A_3_M_2_O_5_]^2+^ layers, including ions of chromium, gallium, vanadium, iron, manganese, scandium, and indium. The smaller Cr^3+^, Ga^3+^ and V^3+^ ions are only found with the 42622 structure, the larger Fe^3+^, Mn^3+^ and Sc^3+^ have been found in both the 32522 and 42622 types, while the largest, In^3+^, has only been found in the 32522 structure. A structure map plotting the average M–O bond length and the average A–O bond length is shown in Fig. 1, this indicates that for a given M–O bond length the 32522 structure has a larger A ion site that the 42622 structure. Therefore, the structure map indicates that novel compositions that might adopt the 32522 structure are more likely to be found in compositions containing larger A ions.

In this work we report on our attempt to identify new materials adopting the 32522 structure with optical and transport properties which will make them viable for p-type conducting or photocatalysis applications. In order to prevent the intra-band trapping states that would be detrimental to both these applications, this requires that the M ion must be a d^0^ and d^10^ trivalent cation.^13^ Our search of this space will include the Sc^3+^ ion, and the less studied larger ions, In^3+^, Y^3+^ and La^3+^ all combined with the largest practicable alkaline earth ion, Ba^2+^, which based on the analysis of the structure map should favour the formation of the 32522 structure. In a systematic review we will report our attempts to combine these four oxide layers; [Ba_3_Sc_2_O_5_]^2+^, [Ba_3_In_2_O_5_]^2+^, [Ba_3_Y_2_O_5_]^2+^, and [Ba_3_La_2_O_5_]^2+^, with the four heavy anion layers; [Cu_2_S_2_]^2−^, [Cu_2_Se_2_]^2−^, [Ag_2_S_2_]^2−^ and [Ag_2_Se_2_]^2−^. This provides a matrix of 16 compounds in which to study the structural and property trends, by XRD, spectroscopy and computational modelling. Four of these sixteen are known, although one has not had a detailed structure published previously. We find that no lanthanum containing compound can be formed, nor any compound with a [Ag_2_S_2_]^2−^ heavy layer, and we will discuss the factors behind the instability of these compositions. However, we can report Ba_3_Sc_2_O_5_Cu_2_Se_2_, Ba_3_Sc_2_O_5_Ag_2_Se_2_, Ba_3_In_2_O_5_Ag_2_Se_2_, and Ba_3_Y_2_O_5_Cu_2_Se_2_ as novel compounds.

## Experimental section

### Solid state synthesis

Layered intergrowth compounds of the form Ba_3_M_2_O_5_M′_2_Ch_2_ were targeted in 0.5 g batches by the conventional ceramic synthesis route – *i.e.* direct combination of appropriate stoichiometric ratios of binary precursors. As shown in eqn (1), for our target layered compounds we combined BaO, BaCh, M_2_O_3_ and M′_2_O in the ratio, 1 : 2 : 1 : 1, where Ch = S or Se; M = Sc, In, Y or La; and M′ = Cu or Ag. The powders were mixed and ground together in a nitrogen-filled glove box, before being pressed into pellets using a hydraulic press with a pressure of 0.75 GPa. The pellets were placed in alumina crucibles and sealed inside a quartz tube under vacuum to form an evacuated ampoule. The samples were reacted at 800 °C for 12 hours, followed by regrinding and resealing under vacuum for a further heat treatment at 800 °C for 12 hours. For eight of the compositions the first two cycles of synthesis failed to yield a single-phase product, a third cycle was attempted at a higher temperature of 900 °C for 72 hours in each case, but this still did not produce the target phase.

1 BaO + 2BaCh + M_2_O_3_ + M′_2_O → Ba_3_M_2_O_5_M′_2_Ch_2_

The metal oxides Sc_2_O_3_, In_2_O_3_, Y_2_O_3_, La_2_O_3_, Cu_2_O (anhydrous) and Ag_2_O were purchased from Sigma-Aldrich with 99.9% purity and used as provided. BaS, BaSe and BaO were all synthesized prior to use as precursors, using the following methods. BaS was synthesized by the decomposition and sulfurization of BaCO_3_ (Alfa Aesar 99.99%) with CS_2_ vapour (Fisher, 99.8%) transported under a flow of argon (BOC Pureshield), at 900 °C for 8 hours.^14^ BaO was synthesized by decomposition of BaCO_3_ under dynamic vacuum at 1000 °C with a dwell time of 14 hours. BaSe was produced from the reduction of BaSeO_4_ under a 5% H_2_ in N_2_ atmosphere (BOC) at 500 °C for 4 hours. The BaSeO_4_ precursor for this reaction was formed by precipitation from aqueous solutions of Na_2_SeO_4_ (Sigma-Aldrich, BioXtra) and Ba(NO_3_)_2_ (Sigma-Aldrich, 99%), over ice. The precipitated BaSeO_4_ was then filtered and dried in air at 70 °C. X-ray diffraction was used to confirm the purity of these in-house synthesised precursors, to within the sensitivity limit of the technique. All precursors and products were stored in a nitrogen filled glove box.

### Characterisation

A Bruker D2 diffractometer equipped with a copper K_α_ X-ray source and Lynx Eye detector was used to collect powder X-ray diffraction patterns, in the range of 10° < 2*θ* < 100°, with a 0.02° step size and a scan time of 3 hours. Collected diffraction patterns were analysed using Rietveld refinement carried out with the GSAS-II software package.^15^ Spectroscopic data were recorded using a PerkinElmer Lambda 750S instrument, equipped with an integrating sphere. Spectra were measured across the range of 300 nm to 2500 nm, and used to determined sample band gaps with the method outlined by Poeppelmeier.^16^

Preliminary photocatalytic studies were conducted on a sample of Ba_3_Sc_2_O_2_Ag_2_Se_2_, using the dichloroindophenol (DCIP) dye degradation test.^17^ A co-catalyst of cobalt oxide was deposited on the oxyselenide powder using a method modified from the literature.^18^ In brief, 25 mg of Ba_3_Sc_2_O_2_Ag_2_Se_2_ was placed in sample vial to which was added 10 mL of a 4.24 mol dm^−3^ Co(NO_3_)_2_·6H_2_O (Sigma Aldrich, 98%) solution in acetone. The acetone was allowed to evaporate overnight, and the cobalt nitrate impregnated powder sample was transferred to an alumina boat which was placed in a tube furnace. The sample was heated to 700 °C under a flow of ammonia, and annealed for 1 hour. The sample was cooled under ammonia flow, and then heated to 200 °C for 1 hour under air exposure. This process initially reduced the cobalt nitrate to metallic cobalt, and then partially oxidised to the desired cobalt oxide co-catalyst, with an approximate loading of 2% cobalt by mass.

The CoO_*x*_@Ba_3_Sc_2_O_2_Ag_2_Se_2_ powder was assessed for photocatalytic activity by taking a 10 mg sample in a sample vial and adding 5 mL of a 6.44 × 10^−5^ M DCIP dye and 2.28 × 10^−3^ M glycerol solution. The test was conducted by placing a crown stirrer in the vial to disperse the powder, and exposing the sample vial to a 5 sun solar simulator (LS0104 150 W Xenon lamp) equipped with a UV filter. At 60 min intervals, for 180 min, an aliquot was taken out of the vial, and then centrifuged to separate the dye from the powder. The visible light absorption spectrum of the dye solution was collected in the range of 300 nm to 800 nm using a PerkinElmer lambda 750S spectrometer. The solution and powder were recombined, and the aliquot returned to the sample vial prior to continued light exposure. Changes in dye concentration caused by photocatalytic degradation were determined using the Beer–Lambert law relationship between absorption and concentration of the dye. Control tests were conducted using dye and glycerol solution under solar simulator illumination without the catalyst present, and the solution with and without catalyst stirred in the dark.

Room temperature conductivity measurements were carried out on a sample of Ba_3_Sc_2_O_2_Cu_2_Se_2_. The compound was pressed into a pellet using a 5 mm die under 2 tons of pressure. The pellet was then annealed in an alumina crucible in a vacuum sealed silica tube at 800 °C for 12 hours. Wire contacts were attached to the annealed pellet (1.5 mm thick, 5 mm diameter) of Ba_3_Sc_2_O_2_Cu_2_Se_2_ using silver epoxy, and four point resistance measurements collected using a Keithley DMM6500 multimeter.

### Computational methodology

First-principles calculations were carried out using the Vienna Ab initio Simulation Package (VASP).^19,20^ Each of the modelled structures was relaxed using the PBEsol functional^21^ with a Hubbard-like *U* correction of 5.17 eV on Cu and Ag.^1^ The Projector-Augmented Wave (PAW) method was used to describe interactions between the core and valence electrons.^22^ A plane-wave cut-off of 550 eV was used to avoid Pulay stress^23^ and a *Γ*-centred mesh with a *k*-point density of at least 70 Å^3^ was used to sample the Brillouin zone. Net forces on ions were reduced to less than 0.01 eV Å^−1^.

Competing phases for all Ba_3_M_2_O_5_M′_2_Ch_2_ compounds were identified using the Materials Project API,^24^ limiting the search to those compounds that appear in the ICSD and resulting in a total of 172 compounds as listed in Table S1 (ESI†). These competing phases were relaxed using the same calculation parameters described above, with a denser *k*-point mesh of at least 100 Å^3^ used for metallic phases. All PBEsol calculations were carried out using the Fireworks python package^25^ and final values for energy above the convex hull of the compositional phase diagram (*E*_hull_) were calculated using the Pymatgen python package.^26^ Bond distances and angles were extracted automatically using the CrystalNN algorithm.^27^

The screened hybrid exchange correlation functional, HSE06,^28,29^ has been shown in previous work to accurately calculate the electronic structure of this family of compounds,^3^ and to calculate accurate band gaps across a wide range of materials,^30^ so was used here to calculate band structures of the stable compounds. A plane-wave cutoff of 550 eV was used and a *Γ*-centred mesh with a *k*-point density of at least 70 Å^3^ was used to sample the Brillouin zone.

Band structures and optical absorption plots were produced using the Sumo python package.^31^ The optical absorption spectra were calculated using the real and imaginary parts of the dielectric constant calculated through a Kramers–Kronig transformation and a summation over the unoccupied bands, respectively, using a method by Adolph and co-workers.^32^ Within this formalism, intra-band and indirect absorptions are not accounted for and only direct valence to conduction band transitions are considered.^33^ Charge carrier effective masses were calculated using the curvature and electronic band extrema:2
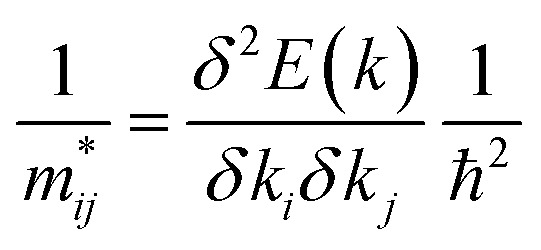
where *E*(*k*) is the energy eigenvalue of the band at *k*-point *k*.

## Results and discussion

We have investigated 32522 compositions of the type Ba_3_M_2_O_5_M′_2_Ch_2_ where M = Sc, In, Y or La; Ch = S or Se; and M′ = Cu or Ag, and can report the successful formation of four novel layered oxychalcogenide phases: Ba_3_Sc_2_O_5_Cu_2_Se_2_, Ba_3_Sc_2_O_5_Ag_2_Se_2_, Ba_3_In_2_O_5_Ag_2_Se_2_ and Ba_3_Y_2_O_5_Cu_2_Se_2_ with a confirmed purity for each of at least 97% as determined by Rietveld refinement of powder XRD data. These materials were annealed twice at 800 °C under vacuum but were found to be air stable once cooled to room temperature, with the exception of Ba_3_Y_2_O_5_Cu_2_Se_2_ which decomposed after several days exposure to air.

### Rietveld refinement

The powder X-ray diffraction patterns of the four novel materials were modelled using the Rietveld method with the structure of the previously reported Ba_3_Sc_2_O_5_Cu_2_S_2_ as a starting point,^3^ using appropriate ion substitutions to match the expected composition in each case. For each phase the lattice, atomic position, and isotropic displacement parameters were refined to optimise the structural model. The sample background was also refined, while the instrumental peak profile parameters were fixed with values derived from refinement of the diffraction pattern of a highly crystalline LaB_6_ sample collected on the same diffractometer. To account for the effect of the sample on Bragg peak broadening, the uniaxial size and strain parameters were refined. Where impurity peaks were identified, variously BaSe, BaCO_3_ and In_2_O_3_, these were modelled using standard crystal structures identified from the inorganic crystal structure database (ICSD),^34–36^ with lattice, particle size, and phase fraction parameters refined.

**Fig. 2:**
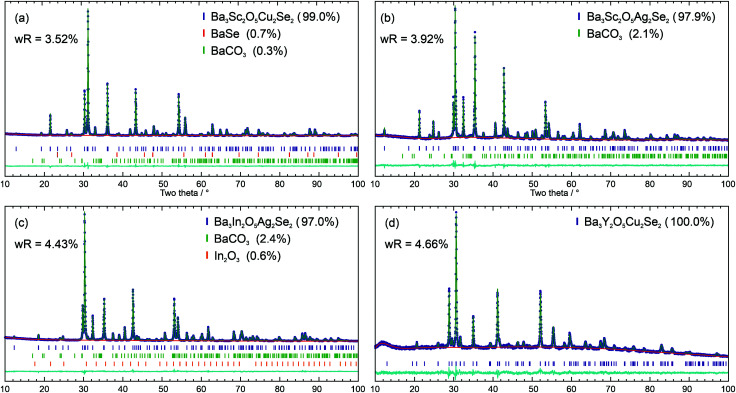
Rietveld refinement fits of the *I*4/*mmm* structure to diffraction data, with purity by mass and w*R*_p_ fit values given for each data set: (a) Ba_3_Sc_2_O_5_Cu_2_Se_2_; (b) Ba_3_Sc_2_O_5_Ag_2_Se_2_; (c) Ba_3_In_2_O_5_Ag_2_Se_2_; (d) Ba_3_Y_2_O_5_Cu_2_Se_2_. In each plot the diffraction data is shown in blue, the model in in green and the background in red. Below the data are tick marks indicating the positions of the peaks, and a cyan difference curve.

Using this approach good fits to the powder diffraction patterns were found for the samples where the observed Bragg peaks could be assigned to the expected target phase, with w*R*_p_ values of less than 4.66% for these four samples. The Rietveld refinement fits to the data are shown in Fig. 2. Ba_3_Sc_2_O_5_Cu_2_Se_2_ was found with 99.0% purity by mass, with the remaining Bragg peaks assigned to small amounts of BaSe and BaCO_3_. The sample of Ba_3_Sc_2_O_5_Ag_2_Se_2_ was 97.9% pure with only BaCO_3_ as an impunity; Ba_3_In_2_O_5_Ag_2_Se_2_ was found alongside 2.4% BaCO_3_ and 0.6% In_2_O_3_; finally, Ba_3_Y_2_O_5_Cu_2_Se_2_ had no identifiable crystalline impurity. A summary of the key refinement fit and lattice parameters for these four samples can be seen in Table 1.

**Table 1:** Summary Rietveld refinement for novel Ba_3_M_2_O_5_M′_2_Ch_2_. All structures were refined in *I*4/*mmm*, and errors are two standard deviations. Full site refinement results and CIF files are available in the ESI

	Ba_3_Sc_2_O_5_Cu_2_Se_2_	Ba_3_Sc_2_O_5_Ag_2_Se_2_	Ba_3_In_2_O_5_Ag_2_Se_2_	Ba_3_Y_2_O_5_Cu_2_Se_2_
Lattice parameter *a*/Å	4.18127(7)	4.21678(11)	4.25908(8)	4.3898(3)
Lattice parameter *c*/Å	27.7132(6)	28.6608(10)	28.8780(6)	27.474(2)
Volume/Å^3^	484.51(2)	509.63(4)	523.84(3)	529.42(9)
Data points	4443	4443	4443	4443
Reflections (main phase)	106	112	112	116
Parameters	37	34	35	46
Purity	99.0%	97.9%	97.0%	100%
w*R*_p_	3.52	3.92	4.43	4.66
RF^2^	2.20	3.76	2.25	4.90
*χ* ^2^	1.80	1.64	1.68	1.41
Colour	Pale brown	Yellow-green	Black	Brown
Ba1 (0.5, 0.5, *z*)	0	0	0	0
Ba2 (0.5, 0.5, *z*)	0.1424(1)	0.1367(1)	0.1384(1)	0.1462(4)
M; (0, 0, *z*)	Sc; *z* = 0.0718(3)	Sc; *z* = 0.0695(3)	In; *z* = 0.0710(1)	Y; *z* = 0.0766(6)
O1 (0.5, 0, *z*)	0.0814(5)	0.0783(5)	0.0804(4)	0.089(2)
O2 (0, 0, *z*)	0	0	0	0
M′; (0.5, 0, *z*)	Cu; *z* = 0.25	Ag; *z* = 0.25	Ag; *z* = 0.25	Cu; *z* = 0.25
Se (0, 0, *z*)	0.198(1)	0.1885(2)	0.1892(1)	0.2002(7)

In addition to the four novel Ba_3_M_2_O_5_M′_2_Ch_2_ compounds discussed above, there are a further four that have been previously reported: Ba_3_Sc_2_O_5_Cu_2_S_2_, Ba_3_InO_5_Cu_2_S_2_, Ba_3_In_2_O_5_Cu_2_Se_2_, and Ba_3_Y_2_O_5_Ag_2_Se_2_, although for the final example only the lattice parameters have been provided, with no further structural details.^3,4,9^ These stable layered oxychalcogenides provide a suite of homologous materials with which to compare our novel compounds, in the discussion below. We did attempt to repeat the synthesis of Ba_3_Y_2_O_5_Ag_2_Se_2_ for this work but found the material to be extremely air sensitive and were unable to collect diffraction data of sufficient quality to refine the atomic positions in order to provide a detailed structural analysis, however we are able to confirm the reported lattice parameters (ESI,† Fig. S1). We found that the related yttrium containing copper selenide, Ba_3_Y_2_O_5_Cu_2_Se_2_, is also air sensitive, but the decomposition is much slower, which allowed us to collect diffraction data of sufficient quality for Rietveld refinement, as described above.

### Lattice parameter trends

**Fig. 3:**
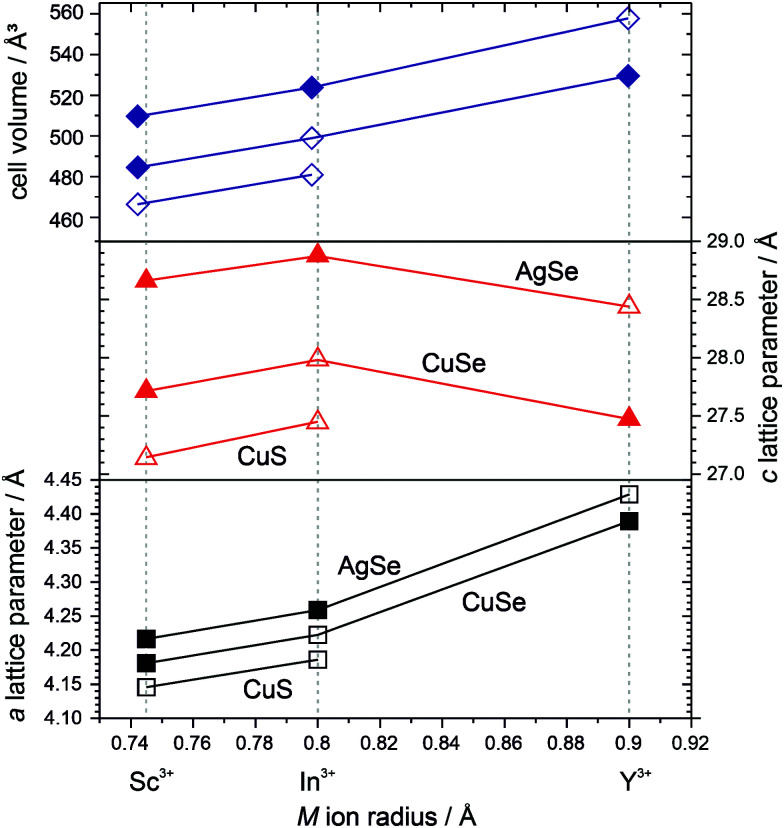
Plot of the lattice parameters for the *I*4/*mmm* structured Ba_3_M_2_O_5_M′_2_Ch_2_ phase plotted against the ionic radius of the M^3+^ ion present in each sample. Guidelines indicate and connect the samples with the same heavy anion layer. The a parameter is shown in the lower graph with squares, and the *c* lattice parameter in the middle graph with triangles./the top graph depicts the cell volume *versus* M ionic radii. Open symbols indicate previously reported materials, from ref. 3, 4 and 9, closed symbols represent novel materials reported in this paper.

Plots of the cell volume and *a* and *c* lattice parameters are shown in Fig. 3, for the four novel and four previously reported layered oxychalcogenides containing the [Ba_3_M_2_O_5_]^2+^ layer, as a function of the ionic radius of the M^3+^ ion. For a given oxide block, increasing the size of the ions in chalcogenide layer also leads to an increase in both *a* and *c* lattice parameter, as would be expected. The magnitude of the change between chalcogenide layers is surprisingly consistent independent of the oxide block in question, an increase of 0.9% in *a* and 2.0% in *c* with substitution of copper sulfide to copper selenide and 0.9% in *a* and 3.3% in *c* with substitution of copper selenide to silver sulfide. Considering the trends for a given chalcogenide layer, we observe the expected increase in the a lattice parameter with increasing M^3+^ ion radius. For example, for the copper selenides, the *a* lattice parameters are 4.18 Å, 4.22 Å and 4.39 Å for samples containing Sc^3+^, In^3+^ and Y^3+^ respectively. In contrast for changes in the *c* lattice parameter something more unusual is observed. There is an increase with the substitution of indium for scandium, but substitution for the larger yttrium ion leads to *a* decrease in the *c* lattice parameter. Overall however, the trends in cell volume are fully consistent with increasing ion size leading to increased cell volume for substitution in either the oxide or chalcogenide layers. Therefore, the unexpected decrease in *c* lattice parameter in Ba_3_Y_2_O_5_Cu_2_Se_2_ and Ba_3_Y_2_O_5_Ag_2_Se_2_ is compensated for by the greater increase in the *a* lattice parameter in each case. To fully explain these trends we must consider the structural changes within each layer.

### Structural details

**Fig. 4:**
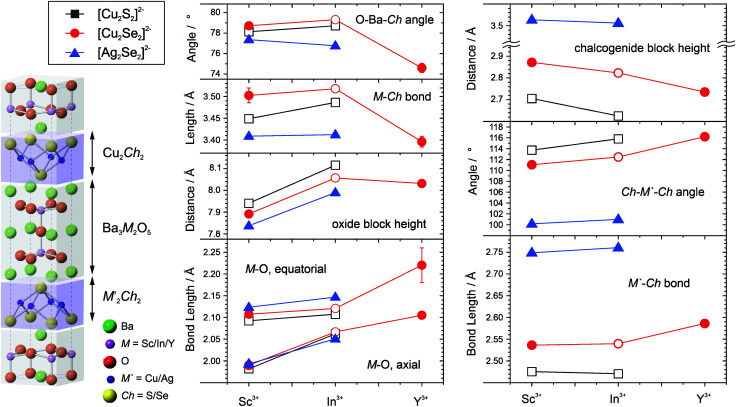
Left schematic diagram of the unit cell of the Ba_3_M_2_O_5_M′_2_Ch_2_; [(M = Sc, In, Y), (M′ = Cu, Ag) and (Ch = S, Se)]. Centre and right plots of the key angles, bond lengths and distances within the structure for the oxide and chalcogenide blocks. Values plotted as a function of the M ion in the oxide block, with chalcogenide layer distinguished by black squares for [Cu_2_S_2_]^2−^, red circles for [Cu_2_Se_2_]^2−^, and blue triangles for [Ag_2_Se_2_]^2−^. Filled symbols are for new materials reported here, empty symbols are from prior literature, ref. 3, 4 and 9. The lines shown connect samples with the same chalcogenide layer as a guide to the eye. Where they are not shown, the error bars are smaller than the symbols used.

Key bond angles, lengths and structural distances are shown in Fig. 4, and tabulated in Table S2 of the ESI.† These can be used to explain the observed changes and trends in the lattice parameters. We observe that for a given oxide block there is an expansion in both the *a* and *c* lattice parameter as a function of the heavy anion layer. This is clearly driven by the increasing length of the chalcogenide bond in order from Cu–S to Cu–Se to Ag–Se, due to the increasing ion sizes. The expansion in the *a* lattice parameter is partially constrained by the static oxide layer, so the longer chalcogenide bond is predominantly accommodated by the flexibility of chalcogenide layer,^3^ with a smaller bond angle and greater chalcogenide block height. This can be observed by consideration of the three samples with the [Ba_3_Sc_2_O_5_]^2+^ layer; with copper sulfide the S–Cu–S bond angle is 114° and the chalcogenide block height is 2.71 Å. The angle tightens to 111° with a block height of 2.87 Å for the copper selenide, the sequence ending with an angle of 100° and block height of 3.51 Å, for the much longer silver selenide bond. The height of the oxide bock, as measured by the Ba–Ba distance, remains relatively constant, varying by only about 0.01 Å from the copper sulfide to the silver selenide as the apical M–O bond length which controls this distance is almost independent of the chalcogenide layer.

For a given chalcogenide layer the expansion in *a* lattice parameter with the replacement of Sc by In is of course driven by the increased size of the M^3+^ ion, which can be seen in the observed increase in the M–O bond length and especially the axial bond lengths, with an increase of 0.08 Å. This leads to the expansion of the oxide block height seen in Fig. 4 in the increase in the Ba–Ba distance. The response of the chalcogenide layer is only a limited change in the chalcogenide bond length, with the flexibly of the layer leading to an increase of the *cis*-bond angle instead, and a correspondingly reduced chalcogenide layer block height. For example, comparing Ba_3_Sc_2_O_5_Ag_2_Se_2_ to Ba_3_In_2_O_5_Ag_2_Se_2_ the silver selenide bond angle increases by 1° and the heavy layer block height decreases by 0.015 Å. Despite this decrease in the height of the chalcogenide layer with exchange of scandium for indium in each of the chalcogenide series, the much larger increase in the height of the oxide layer predominates and accounts for the observed increase in *c* lattice parameter.

These trends in bond length and angle changes in the oxide and chalcogenide blocks extend to Ba_3_Y_2_O_5_Cu_2_Se_2_, yet despite this we observe the anomalous decrease in the *c* lattice parameter. This can be rationalised as the decrease in the cell height comes from a significant drop in the interlayer spacing controlled by the square cuboid environment of the interlayer barium. The significant expansion in the *a* lattice parameter, when comparing Ba_3_In_2_O_5_Cu_2_Se_2_ to Ba_3_Y_2_O_5_Cu_2_Se_2_, caused by the much larger yttrium ion results in a flattening of the cuboid barium environment, as seen in the sharp decrease in the O–Ba–Ch angle from 79.3° to 74.6°, and a significant decrease in the interlayer spacing, as measured by the M*–*Ch distance which spans this environment and accounts for a 0.49 Å decrease across the unit cell. This can be thought of as the chalcogenide and oxide layers expanding in the basal direction around the inter-layer barium and towards each other in the 001 direction, as the barium ion is no longer the ideal size for the site when compared to the pairings with smaller ions.

### DFT calculations

DFT calculations were carried out for all of the 16 proposed Ba_3_M_2_O_5_M′_2_Ch_2_ compositions, and their stability compared to binary and ternary competing phases. Full details of the results of these calculations can be found in Table S3 (ESI†). Eight of these were found to have an energy above the convex hull (*E*_hull_) of zero, indicating that they are stable compared to the competing phases. These eight stable compounds matched with the four previously reported samples and the four novel compounds prepared here. The eight compounds determined to be unstable with respect to the competing phases were those that also could not be experimentally prepared, which will be discussed in more detail below. Overall, it highlights for these layered oxychalcogenide systems that energy above the convex hull is an excellent predictor for experimental stability.

The calculations also allowed the structures to be predicted, including the material for which we could not refine a structure, Ba_3_Y_2_O_5_Ag_2_Se_2_. There was a good match between the structures derived from Rietveld modelling of experimental data and the structures derived from computational methods, with the lattice parameters of the models matching experimental results within 1% for all samples. Comparing the calculated bonds and angles, the observed differences between the two models were: barium–barium distance, less than 0.4%, barium to anion bonds less than 2%, M–O bonds <1.5% and O–M–O angle <2% (M = Sc, In, Y). Previous work has shown that the PBEsol functional slightly overestimates the chalcogenide bond angle compared to experiment, and this is reflected again here with both copper and silver chalcogenide angle. The computational model over predicts the Ch–M′–Ch angle by up to 3.5%, and underpredicts the bond length by 3%.

Overall, the accurate match between the DFT calculations and the empirically derived structural models provides confidence that we can reliably interpret the model derived for Ba_3_Y_2_O_5_Ag_2_Se_2_ for which it was not possible to collect Rietveld quality data. For Ba_3_Y_2_O_5_Ag_2_Se_2_, the DFT structural model indicates a continuation of the structural trends observed in the rest of the series. The reduction in the length of *c* parameter compared to Ba_3_In_2_O_5_Ag_2_Se_2_, which we can observe experimentally, is due to a significant decrease in the inter-layer spacing, as the expansion in the *a* lattice parameter driven by the larger yttrium ion forces a flattening of the square cuboid environment of the inter layer barium. There is almost no expansion in the oxide block in the 001 direction despite the larger yttrium cation, and only the expected slight decrease in the chalcogenide block height.

### Electronic structure and optical properties

**Fig. 5:**
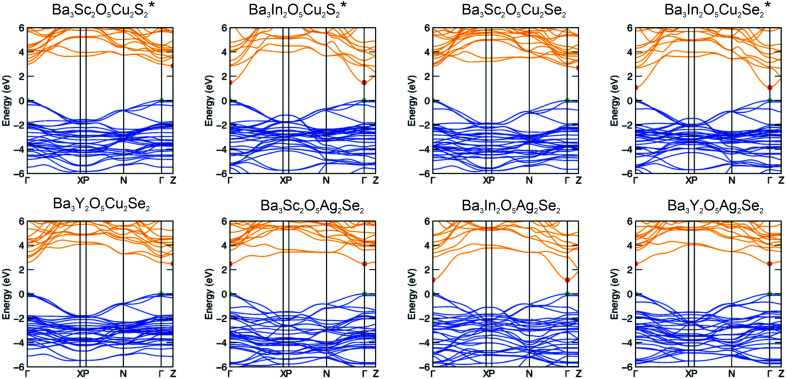
Electronic band structures of the eight thermodynamically stable Ba_3_M_2_O_5_M′_2_Ch^2^ compounds calculated using the HSE06 DFT functional. Red and green dots mark the conduction band minimum (CBM) and valence band maximum (VBM), respectively. Compositions marked with an asterisk have been published previously.^3,4^

The full electronic band structures of the eight stable compounds are shown in Fig. 5 and these are used to calculate the light hole effective masses (m^*^_hole_). Three of these band structures have been previously reported (Ba_3_Sc_2_O_5_Cu_2_S_2_, Ba_3_In_2_O_5_Cu_2_S_2_ and Ba_3_In_2_O_5_Cu_2_Se_2_) and are reproduced here for completeness, marked with an asterisk.^3,4^ The values of m^*^_hole_ in the [Cu_2_Ch_2_]^2−^/[Ag_2_Se_2_]^2−^ planes are found at *Γ*–*X* and *Γ*–*N* and are relatively low. The values for each sample are reported in Table 2, but across all samples the intraplane hole masses are found in the range 0.37 *m*_e_ to 0.65 *m*_e_, with lower values for selenide containing materials and higher values for the sulfide compounds. Overall the hole masses are comparable to the values predicted for Sr_3_Sc_2_O_5_Cu_2_S_2_ of 0.35 *m*_e_ to 0.45 *m*_e_,^1^ and in which high hole mobility has been experimentally verified. The *Γ*–*Z* reciprocal space direction corresponds to hole transport in the interplanar direction, and the modelled band structures show very flat bands, indicating much higher hole masses and limited mobility, as expected.

**Table 2:** Calculated and experimental band gaps, with calculated light hole mass ranges. The orbitals comprising the VBM and CBM are also listed. Samples are listed in order of decreasing band gap size

	Band gap/eV	HSE06 optical band gap/eV	Light hole mass/*m*_e_	VBM composition	CBM composition
Ba_3_Sc_2_O_5_Cu_2_S_2_^a^	3.27^a^	3.24	0.6–0.64	S 2p	Ba 5d, Cu 4s
Ba_3_Y_2_O_5_Cu_2_Se_2_	b	3.04	0.46–0.5	Se 3p	Ba 5d, Cu 4s
Ba_3_Sc_2_O_5_Cu_2_Se_2_	3.09	2.95	0.45	Se 3p	Ba 5d, Cu 4s
Ba_3_Y_2_O_5_Ag_2_Se_2_^a^	b	2.63	0.46–0.48	Se 3p	Ag 5s
Ba_3_Sc_2_O_5_Ag_2_Se_2_	2.58	2.60	0.44–0.56	Se 3p	Ag 5s
Ba_3_In_2_O_5_Cu_2_S_2_^a^	1.77^a^	1.84	0.54–0.56	S 2p	In 5s
Ba_3_In_2_O_5_Ag_2_Se_2_	1.44	1.58	0.37–0.56	Se 3p	In 5s
Ba_3_In_2_O_5_Cu_2_Se_2_^a^	1.32^a^	1.48	0.40–0.49	Se 3p	In 5s

a Previously reported.

b Uncertainty due to sample air sensitivity. Light hole effective masses for the newly reported compounds are broken down further by reciprocal space direction in Table S5 (ESI).

**Fig. 6:**
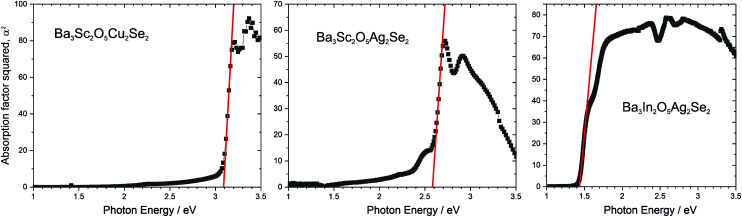
Plots of *α*^2^ against photon energy for novel compositions, Ba_3_Sc_2_O_5_Cu_2_Se_2_, Ba_3_Sc_2_O_5_Ag_2_Se_2_, and Ba_3_Sc_2_O_5_Ag_2_Se_2_, to allow band gaps to be determined from tangents shown in red. Absorption co-efficient determined using the Kubelka–Munk transformation. Spectra for yttrium containing samples could not be obtained due to air sensitivity.

The band gaps of the novel materials were experimentally determined from analysis of the diffuse reflection spectra, using the method recently outlined by Poeppelmeier, as suitable for crystalline, non-degenerate semiconductors.^16^ In brief, the Kubelka–Munk function was used to estimate the sample absorption from the reflectivity,^37^ and then a plot of the square of the absorption against the photon energy was used to extrapolate a tangent from the point of inflection to determine the band gap of the sample (Fig. 6). This was carried out for three of the novel materials, Ba_3_Sc_2_O_5_Cu_2_Se_2_ (band gap of 3.1 eV), Ba_3_Sc_2_O_5_Ag_2_Se_2_ (2.6 eV), Ba_3_In_2_O_5_Ag_2_Se_2_ (1.4 eV). These band gaps values are given in Table 2, alongside those previously reported for Ba_3_Sc_2_O_5_Cu_2_S_2_, Ba_3_In_2_O_5_Cu_2_S_2_ and Ba_3_In_2_O_5_Cu_2_Se_2_ and the values determined computationally from calculated optical absorption plots (Fig. S2, ESI†). For the yttrium containing samples experimental measurements could not be made due to their air sensitivity, so only the modelled band gaps are reported. Overall, there is good agreement between the experimental and calculated band gaps, as would be expected based on what we have shown previously for this family of materials.^3^

The trends in the band gaps observed for this series of compounds can be explained by considering the composition of the band edges derived from the electronic structure modelling. For all of the compounds, the valence band maximum (VBM) is comprised predominantly of the filled chalcogenide p orbitals, while the conduction band minimum (CBM) is predominantly composed of the empty coinage metal s states, except for the indium containing samples with the [Ba_3_In_2_O_5_]^2+^ block, where the In 5s states dominate then CBM. Ignoring the for the moment indium containing samples, this means that in the majority of the samples the size of the band gap is dependant only on the composition of the chalcogenide layer. We would expect sulfides to have larger band gaps than selenides, as the more electronegative sulfur lowers the VBM, while compounds with copper in the heavy layer would have a larger band gap than silver containing samples, as the more electronegative silver lowers the position of the CBM.

This explains why Ba_3_Sc_2_O_5_Cu_2_S_2_ has the largest band gap (3.24 eV, modelled value), due to the electronegative sulphur lowering the VBM, and the relatively electropositive copper raising the CBM. As both Ba_3_Y_2_O_5_Cu_2_Se_2_ and Ba_3_S_2_O_5_Cu_2_Se_2_ share the copper selenide heavy layer they have similar modelled band gaps (3.04 eV and 2.95 eV), due the VBM and CBM being composed of the same orbitals, but smaller than Ba_3_Sc_2_O_5_Cu_2_S_2_ due to the contribution of the more electropositive selenium to the VBM. The silver selenides; Ba_3_Y_2_O_5_Ag_2_Se_2_ and Ba_3_S_2_O_5_Ag_2_Se_2_ both have smaller band gaps of 2.63 eV and 2.60 eV, due to presence of the lower lying silver 5s states. The indium containing compounds have much smaller band gaps, as indium 5s states are much lower in energy than either copper or silver ns states giving band gap energies of 1.84 eV, 1.58 eV and 1.48 eV for the copper sulfide, silver selenide and copper selenide, respectively. The small differences observed in the modelled band gaps for the pairs Ba_3_Sc_2_O_5_Cu_2_Se_2_ and Ba_3_Y_2_O_5_Cu_2_Se_2_ (2.95 eV to 3.04 eV); Ba_3_Sc_2_O_5_Cu_2_Se_2_ and Ba_3_Y_2_O_5_Cu_2_Se_2_ (2.60 eV to 2.63 eV); and Ba_3_In_2_O_5_Cu_2_Se_2_ and Ba_3_In_2_O_5_Ag_2_Se_2_ (1.48 eV to 1.58 eV), despite each pair having identical band edge compositions, is due to a secondary effect where an increase in the basal lattice parameter reduces overlap and decreases the dispersion in the bands leading to the slight increase in band gap.^8^

From the novel compounds reported in this work we can conclude from the electronic structure data that Ba_3_Sc_2_O_5_Cu_2_Se_2_ is of most interest as a possible p-type transparent conductor. It has a calculated hole mobility equivalent to Sr_3_Sc_2_O_5_Cu_2_S_2_, and a sufficient large band gap to prevent visible light absorption and is air stable. We can also conclude that Ba_3_Sc_2_O_5_Ag_2_Se_2_ might be a functional visible light photocatalyst, based on its reasonable transport properties and 2.6 eV band gap.

The visible light photocatalytic potential of Ba_3_Sc_2_O_5_Ag_2_Se_2_ was assessed using a dye degradation test, where a solution of dichloroindophenol (DCIP) dye undergoes reductive decolouration in the presence of glycerol as a sacrificial oxidant, enhanced by the photocatalyst. This is an effective and simple test to assess visible light photocatalysts.^17^ The sample of Ba_3_Sc_2_O_5_Ag_2_Se_2_ was loaded with a cobalt oxide co-catalyst to enhance its activity,^18^ and allow for measurements to be conducted in a 3 hour time window. A 5 sun solar simulator with a UV light filter was used as the light source. This found that 10 mg of CoO_*x*_@Ba_3_Sc_2_O_5_Ag_2_Se_2_ in 5 mL of 6.44 × 10^−5^ M dye solution was able to degrade 15.1% of the dye during the three hour experiment. No degradation of the dye was observed without the catalyst present, or in the presence of the catalyst without the light source. This confirms that Ba_3_Sc_2_O_5_Ag_2_Se_2_ is a visible light photocatalyst, comparable to other layered oxychalcogenides tested using the same procedure, with a similar rate to Ba_3_In_2_O_5_Cu_2_S_2_ (14.5% dye degradation in three hours), but lower than Ba_3_Sc_2_O_5_Cu_2_S_2_ (27.0% degradation).^4^

Additionally, room temperature conductivity measurements were conducted on a pellet of Ba_3_Sc_2_O_5_Cu_2_Se_2_, which was found to have a density of 73% relative to the theoretical density derived from diffraction data. Four point probe measurements found that the pellet had a conductivity of 5.0(1) × 10^−5^ S cm^−1^, which is comparable to previous reports on other promising for pristine undoped layered oxychalcogenides such as LaOCuS (1 × 10^−4^ S cm^−1^),^10^ and Sr_2_GaO_3_CuS (2.2 × 10^−4^ S cm^−1^),^38^ although not as high as values reported for LaOCuSe (24 S cm^−1^) or Sr_3_Sc_2_O_5_Cu_2_S_2_ (2.8 S cm^−1^).^2,39^ However, it should also be considered that the value reported here for Ba_3_Sc_2_O_5_Cu_2_Se_2_ are for an annealed pellet, rather than a plasma sintered one.

### Discussion of the unstable compositions

Above we discussed in detail the structural and electronic properties and relationships of the eight compounds that were found to be stable and could be synthesised from the 16 possible Ba_3_M_2_O_5_M′_2_Ch_2_ compounds, where M = In, Sc, Y, Ga and M′ = Cu, Ag. It is valuable to consider, however, the factors which underlie why the remaining eight materials are not stable.

We have not been able to form any compound containing lanthanum, *i.e.* those with a [Ba_3_La_2_O_5_]^2+^ layer. All Bragg peaks observed in the diffraction patterns of our attempts at these samples could be indexed to elemental or binary by-products, as summarised in Table S4 (ESI†), with no indication of the expected peaks of the target phases. These syntheses were speculative, as there are no prior examples of lanthanum in the 5 coordinate M^3+^ ion environment in the 32522-structure type. The lanthanum ion, with an ionic radius of 1.03 Å, is considerably larger than the yttrium ion (0.90 Å) which is the largest ion found on the M site in the known examples of the structure type. We hypothesise that the limiting size for ions in the oxide block can be predicted based on the Goldschmidt tolerance factor, *t*, originally derived for predicting the formability of the perovskite structure, using the relationship of the relative ion sizes *r*_A_, *r*_B_ and *r*_O_ for the 12 coordinate cation site, the 6 coordinate cation site and the anion site, as shown in eqn (3). Although not always a guarantee of stability, it is successful as a filter for unstable compositions in the perovskites.^40^ A survey of all of the known 32522 structure types (and related 42622 structure) finds that these all have oxide layers with tolerance factors in the range of 0.9 to 1.0, which matches that observed for undistorted, cubic perovskites, implying that these oxychalcogenide structure types have a relative tight tolerance for the size of the ions. Calculating the tolerance factor for [Ba_3_La_2_O_5_]^2+^ give a value of 0.88, which falls outside of this stable range, and indicates that the barium ion is too small for the lanthanum ion. The tolerance factors for the oxide layers can be found in Table 3. Perovskite type compounds can be made with tolerance factors less than 0.9, but these undergo distortion through polyhedral tilting, to reduce the size of the 12-coordinate A site. It is likely that such distortions are not possible in the 32522 structure due to the need for commensurate bonding to the chalcogenide layer.3
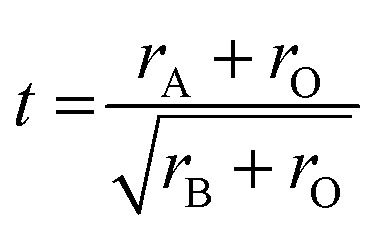
The computational modelling for the lanthanum compounds also predicted them to be unstable with respect to decomposition. However, the relaxed structural models were produced assuming that the decomposition did not occur and, within the symmetry limitations, in order to accommodate the lanthanum ion these would have to have *a* lattice parameters of 4.49 Å for the copper sulfide, increasing to 4.56 Å for the silver selenide, values larger than any observed for a known layered oxychalcogenide. Inspection of the structural model for these found the central barium atom to be significantly under-bonded. This barium should be in a 12 co-ordinate geometry, however the length of the apical Y–O bond increases the size of the 12 co-ordinate site such that the barium is in effect only bonded to the equatorial oxygen ions providing an under-bonded pseudo-square planar geometry. Overall the computational modelling of the proposed lanthanum containing structures confirm that these are unstable due to the large size of the lanthanum ion being incompatible with the size of the barium and also the majority of the chalcogenide layers.

**Table 3:** Tolerance factors for the oxide layers considered in this work

Oxide layer	Tolerance factor, *t*
[Ba_3_Sc_2_O_5_]^2+^	0.99
[Ba_3_In_2_O_5_]^2+^	0.97
[Ba_3_Y_2_O_5_]^2+^	0.93
[Ba_3_La_2_O_5_]^2+^	0.88

We were also unable to experimentally synthesise any compound containing a silver sulfide layer, nor were any predicted to be stable from computational methods. At first this is surprising, given the ability to synthesis examples of all the other copper and silver chalcogenide layers. However, in each of the attempts at forming a silver selenide, the same by-products were observed, elemental silver and barium sulphate. Our hypothesis for this instability is that silver is too readily reduced by sulfide, with BaSO_4_ acting as a thermodynamic sink. In contrast the silver selenides are stable because of the lower reduction potential of Se^2−^ compared to S^2−^, and the consequent lower enthalpy of formation of BaSeO_4_. This also helps to explain the lack of prior examples of layered silver sulfides in the literature, where to our knowledge there are only two examples, these are LaOAgS,^41^ and Sr_3_Fe_2.5_O_5_Ag_1.5_S_2_; in the former there is no alkaline earth to form a sulphate and in the latter the composition is stabilised by partial substitution of silver for iron in the sulfide layer.^42^

The final compound that could not be synthesized was Ba_3_Y_2_O_5_Cu_2_S_2_. In our attempt at the synthesis of this we could not identify any of the expected peaks of the target phase in the diffraction pattern collected on the sample, which instead was found to contain Bragg peaks which could indexed to a mixture of Y_2_O_3_, BaS, Cu, BaSO_4_ and BaYO_4_. This inability to combine the [Ba_3_Y_2_O_5_]^2+^ layer with the [Cu_2_S_2_]^2−^ layer can be rationalised based on the expected size. Yttrium is the largest of the three ions considered in the oxide block, while the [Cu_2_S_2_]^2−^ layer contains the smallest paring of ions for the chalcogenide layer, so it is likely that the [Ba_3_Y_2_O_5_]^2+^ is simply too large to be accommodated in the structure with the copper sulfide layers. Although predicted to be unstable, it was still possible to computationally model and produce a relaxed structure for Ba_3_Y_2_O_5_Cu_2_S_2_, which had predicted lattice parameters of 4.34 Å and 26.48 Å. The largest previously reported *a* lattice parameter for a layered oxychalcogenide containing copper sulfide layers is the value of 4.18 Å in Ba_3_In_2_O_5_Cu_2_S_2_.^4^ In order to achieve this theoretical basal expansion to 4.34 Å the model requires an extreme stretching of the copper sulfide geometry, such that the S–Cu–S angle becomes 125°, even accounting for the tendency to over-predict the angle value, this is an extreme distortion from the ideal tetrahedral geometry. This would support the argument of size incompatibility as the reason for the inability to synthesis Ba_3_Y_2_O_5_Cu_2_S_2_, and also suggest the possible upper size limit for inclusion of the copper sulfide layer is approximately 4.20 Å and certainly no larger than 4.35 Å.

## Conclusions

Our investigation of the Ba_3_M_2_O_5_M′_2_Ch_2_ compositions with coinage metal chalcogenide layers, and scandium, indium, yttrium and lanthanum oxide layers through both modelling and synthesis, has allowed us to identify four new compositions, Ba_3_Sc_2_O_5_Cu_2_Se_2_, Ba_3_Sc_2_O_5_Ag_2_Se_2_, Ba_3_In_2_O_5_Ag_2_Se_2_, and Ba_3_Y_2_O_5_Cu_2_Se_2_, and confirm that by targeting larger ions in the oxide layer we can promote the formation of this 32522 structure. We have identified that the Goldschmidt tolerance factor can be used as a simple guide for predicting formability, and that this must lie within a range of 0.9 to 1.0 for ions in the oxide layer in order to produce a stable 32522 structure. Additionally, computational modelling is highly effective at predicting stability for this series of compounds. We have also provided an explanation for the instability of layered oxychalcogenides with silver sulfide layers, which is due to the favourable redox reaction between sulfide and silver ions. Finally, we have identified that Ba_3_Sc_2_O_5_Ag_2_Se_2_ has potential as visible light photocatalyst, based on its transport properties and band gap, while Ba_3_Sc_2_O_5_Cu_2_Se_2_ is of interest as a possible p-type transparent conductor, and confirmed these with preliminary experiments.

## Conflicts of interest

There are no conflicts to declare.

## Supplementary Material

TC-010-D1TC05051F-s001
