# Correction: Investigation of factors affecting the stability of compounds formed by isovalent substitution in layered oxychalcogenides, leading to identification of Ba_3_Sc_2_O_5_Cu_2_Se_2_, Ba_3_Y_2_O_5_Cu_2_Se_2_, Ba_3_Sc_2_O_5_Ag_2_Se_2_ and Ba_3_In_2_O_5_Ag_2_Se_2_

**DOI:** 10.1039/d3tc90249h

**Published:** 2023-11-29

**Authors:** Gregory J. Limburn, Daniel W. Davies, Neil Langridge, Zahida Malik, Benjamin A. D. Williamson, David O. Scanlon, Geoffrey Hyett

**Affiliations:** a School of Chemistry, University of Southampton Southampton SO17 1BJ UK g.hyett@soton.ac.uk; b Department of Chemistry, University College London 20 Gordon Street London WC1H 0AJ UK; c Research Computing Service, Information and Communication Technology, Imperial College London London SW7 2AZ UK; d Department of Materials Science and Engineering, Norwegian University of Science and Technology (NTNU) Trondheim 7491 Norway

## Abstract

Correction for ‘Investigation of factors affecting the stability of compounds formed by isovalent substitution in layered oxychalcogenides, leading to identification of Ba_3_Sc_2_O_5_Cu_2_Se_2_, Ba_3_Y_2_O_5_Cu_2_Se_2_, Ba_3_Sc_2_O_5_Ag_2_Se_2_ and Ba_3_In_2_O_5_Ag_2_Se_2_’ by Gregory J. Limburn *et al.*, *J. Mater. Chem. C*, 2022, **10**, 3784–3795; https://doi.org/10.1039/D1TC05051F.

The authors regret an error in the published article title and abstract, where the formula Ba_3_Y_2_O_5_Cu_2_Se_2_ was incorrectly given as Ba_3_Y_2_O_5_Cu_2_S_2_. All other occurrences of the formula throughout the published article were shown correctly. The corrected article title is as shown here.

The Royal Society of Chemistry apologises for these errors and any consequent inconvenience to authors and readers.

## Supplementary Material

