# Supplementary material for: Investigation of factors affecting the stability of compounds formed by isovalent substitution in layered oxychalcogenides, leading to identification of Ba3Sc2O5Cu2Se2, Ba3Y2O5Cu2S2, Ba3Sc2O5Ag2Se2 and Ba3In2O5Ag2Se2
Source: J Mater Chem C Mater. 2022 Feb 8;10(10):3784–95. doi: 10.1039/d1tc05051f (PMC9558239; doi:10.1039/d1tc05051f)
Supplement: TC-010-D1TC05051F-s001 [file TC-010-D1TC05051F-s001.pdf]

Electronic Supplementary Material (ESI) for Journal of Materials Chemistry C.
This journal is © The Royal Society of Chemistry 2022

ELECTRONIC SUPPLEMENTARY INFORMATION

Investigation of Factors Affecting the Stability of Compounds Formed by Isovalent Substitution in Layered Oxychalcogenides, Leading to Identification of $Ba_3Sc_2O_5Cu_2Se_2$, $Ba_3Y_2O_5Cu_2S_2$, $Ba_3Sc_2O_5Ag_2Se_2$ and $Ba_3In_2O_5Ag_2Se_2$.

Gregory J. Limburn[a], Daniel W. Davies[b,c], Neil Langridge[a], Zahida Malik[a], Benjamin A. D. Williamson[c], David O. Scanlon[b], Geoffrey Hyett[a]*

[a] School of Chemistry, University of Southampton, Southampton, SO17 1BJ, UK
[b] Department of Chemistry, University College London, 20 Gordon Street, London, WC1H 0AJ, UK
[c] Research Computing Service, Information and Communication Technology, Imperial College London, London, SW7 2AZ, UK.
[d] Department of Materials Science and Engineering, Norwegian University of Science and Technology (NTNU), Trondheim 7491, Norway
*Corresponding Author: g.hyett@soton.ac.uk

	Formula	ΔHf / eV	Formula	ΔHf / eV	Formula	ΔHf / eV
0	CuS	-3.44547	Se	-3.8054	$BaSe_2$	-4.67216
1	$Ba(Ag_3O_2)_2$	-3.61945	$La_{10}S_{14}O$	-7.15085	In	-2.98475
2	$Ba2Y(CuO_2)_4$	-5.93288	Cu	-2.59161	$Y_2(SO_4)_3$	-7.49282
3	$BaScCuS_3$	-5.7897	$Sc_2Se_3O_{10}$	-6.86857	Y_2S_3	-7.49454
4	CuS_2	-3.97707	In_4Ag_9	-2.10105	$LaCu_2$	-3.6207
5	$Ba_2LaAg_5S_6$	-4.29224	Y_2O_3	-9.51497	$BaIn_2$	-3.23909
6	$LaCuO_3$	-6.76352	$ScSe$	-6.89272	Ba_4InAgS_6	-4.8908
7	$BaAg_8S_5$	-3.10055	$AgSO_4$	-5.4649	LaS_2	-6.63879
8	$Ba_{11}In_6O_3$	-3.91582	$BaLaCuS_3$	-5.66111	$Ba_2(CuO_2)_3$	-5.3824
9	$ScCu_2$	-4.09059	$CuSeO_4$	-5.13146	$BaCu_4S_3$	-3.98347
10	$Ba(AgS)_2$	-3.80156	Ag_2S	-2.66386	$Ba(YS_2)_2$	-6.95337
11	$Ba_4Y(CuO_3)_3$	-6.19579	$Ag_2S_2O_7$	-5.50571	Ag_2SeO_4	-4.55251
12	$La_3CuS_3O_2$	-7.17984	Y	-6.81909	$Ba(InS_2)_2$	-4.75879
13	Sc_2Se_3	-6.67668	$ScCu$	-4.77195	$Ba(InSe_2)_2$	-4.37299
14	Sc_2SO_2	-8.72514	Se_2O_5	-5.37804	$ScAgO_2$	-7.00477
15	CuO	-4.34392	$LaSO$	-7.68492	In_2O_3	-5.96473
16	$BaSe$	-5.03127	$CuSe_2O_5$	-5.25636	$LaCu_5$	-3.12038
17	InS	-4.2109	$BaLaCuSe_3$	-5.24417	Y_2Se_3	-6.94221
18	$BaCu_{13}$	-2.54684	$Ba_2In_2S_5$	-4.90478	La_2SeO_2	-8.13889
19	BaS_3	-4.95371	$BaYCuSe_3$	-5.4821	La_2SO_6	-7.95241
20	$LaAg_2$	-3.06689	$BaYAgSe_3$	-5.28202	O_2	-5.14269
21	$In_2(SO_4)_3$	-6.31404	$LaSO_5$	-7.18884	$Ba_2In_2O_5$	-6.12921
22	$BaIn_4$	-3.21708	Sc_2S_3	-7.23503	Ag	-1.64606
23	$LaCu_{13}$	-2.8278	LaS	-7.09282	$LaSe$	-6.62074
24	$Sc_2(SeO_4)_3$	-6.67837	$BaCu$	-2.38677	BaO	-6.20053
25	Y_5S_7	-7.55252	$YAgSe_2$	-5.30327	$La_2Cu(SeO_3)_4$	-6.42915
26	ScS	-7.47639	Ag_2Se	-2.54178	$LaCuSe_2$	-5.29228
27	$LaCuS_2$	-5.72764	Ag_3O_4	-3.69829	S	-4.39088
28	$Ba_3In_2O_6$	-6.15612	La	-5.3462	$La_{10}S_{19}$	-6.70236
29	$BaCu(SeO_3)_2$	-5.69116	Ba_2CuO_3	-5.65774	$In_2Se_2O_7$	-5.7523
30	$LaCuO_2$	-6.95227	$Sc_2(SeO_3)_3$	-6.95932	Ba_9In_4	-2.70645
31	$LaCuSeO$	-6.35921	SO_3	-6.1372	$BaSeO_4$	-6.20327
32	YAg_2	-3.58113	Cu_2O	-3.95268	BaS_2	-5.08926
33	$BaAg_5$	-1.81549	La_2CuS_4	-6.02146	La_2O_3	-8.7395
34	$YCuS_2$	-6.01493	La_2SO_2	-8.31701	$ScAg$	-4.29051
35	YSe	-7.18956	In_3Cu_7	-2.75803	$ScCuO_2$	-7.34865
36	Cu_7S_4	-3.5266	S_8O	-4.61868	In_5AgSe_8	-3.85558
37	$LaCu$	-4.04102	$BaYCuS_3$	-5.90815	$InCuSe_2$	-3.78007
38	In_2S_3	-4.40103	Ag_2SO_4	-5.08389	$InAg_3$	-2.01434
39	$ScAg_4$	-2.76475	$LaSe_2$	-6.14021	$InAgS_2$	-3.82866
40	$BaSO_4$	-6.88254	$InCuS_2$	-4.13963	Ag_2O	-2.95711
41	$CuSe$	-3.07064	$InAgO_2$	-4.8634	AgO	-3.51608
42	YCu_5	-3.42332	$ScCuS_2$	-5.87132	$BaSeO_3$	-6.26115
43	$La_4Se_3O_4$	-7.79831	$In_2(Se_2O_5)_3$	-5.63275	$LaCu_6$	-3.06584
44	$BaAg_2$	-2.00915	$BaIn$	-3.02891	$La(CuO_2)_2$	-6.14583
45	$InAgSe_2$	-3.50654	$La_{10}Se_{14}O$	-6.66596	Cu_3Se_2	-3.29692
46	Ag_2SeO_3	-4.39893	YCu_2	-4.18365	$Y(CuO_2)_2$	-6.46098
47	$BaSc_2O_4$	-8.53885	$CuSO_4$	-5.76258	BaY_2O_4	-8.59603
48	YAg	-4.44208	Sc_2O_3	-9.39222	$BaAg$	-2.07645
49	$LaCuSO$	-6.54693	$CuSe_2$	-3.5552	$Ba(YSe_2)_2$	-6.45466
50	$ScAg_2$	-3.49669	$La_2(SeO_3)_3$	-6.876	YCu	-4.87659
51	Sc	-6.60553	$BaSc_3AgS_6$	-6.33319	La_2CuO_4	-7.50879
52	BaO_2	-6.10381	$La_{10}Se_{19}$	-6.20415	BaS	-5.35968
53	$LaAg$	-3.61635	$YAgO_2$	-7.06044	$Ba(CuO)_2$	-4.94118
54	$InSe$	-3.89542	BaS_4O_{13}	-6.42946	Ba	-2.14891
55	Y_2SO_2	-8.9782	SO_2	-5.99127	$BaSe_2O_5$	-6.00657
56	YS	-7.73259	$BaLaAgSe_3$	-5.06875		
57	La_2S_3	-7.00169	SeO_2	-5.48985		

Table S1. List of competing phases used to calculate energy above hull for each of the target phases.

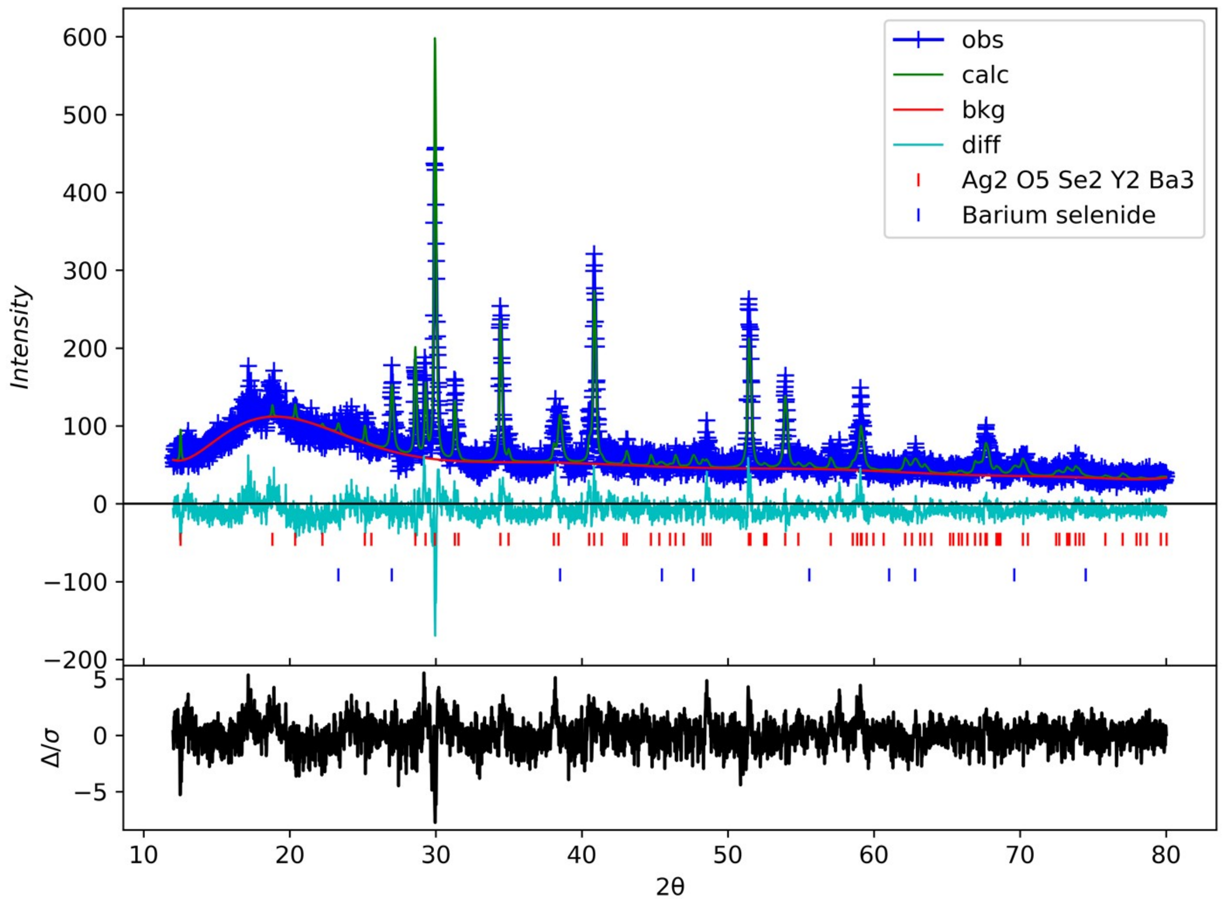

Figure S1. Diffraction pattern of the re-attempted synthesis of $Ba_3Y_2O_5Ag_2Se_2$, previously reported by Ogino et al. Pattern collected over 10 mins, longer scan times lead to appearance of degradation products due to air exposure. Data of insufficient quality to allow for refinement of structural parameters. However, a simplified Rietveld refinement was conducted, assuming the atomic positions matched house found in homologous $Ba_3Y_2O_5Cu_2Se_2$. This refinement confirmed the lattice parameters for $Ba_3Y_2O_5Ag_2Se_2$ as a = 4.42 Å and c = 28.38 Å.

Bond or Angle	$Ba_3Sc_2O_5Cu_2Se_2$	$Ba_3Sc_2O_5Ag_2Se_2$	$Ba_3In_2O_5Ag_2Se_2$	$Ba_3Y_2O_5Cu_2Se_2$
O-Ba-*Ch* angle / °	78.7(5)	77.4(2)	76.8(2)	74.6(9)
M-*Ch* Bond / Å	3.503(34)	3.409(10)	3.413(4)	3.396(25)
Ba-Ba distance, Oxide Block height / Å	7.892(5)	7.837(6)	7.990(4)	8.032(24)
M-O equatorial bond / Å	2.107(2)	2.123(2)	2.147(2)	2.220(80)
M-O axial bond /Å	1.990(8)	1.993(8)	2.051(2)	2.105(16)
Ch Block height Å	2.872(35)	3.528(6)	3.512(4)	2.735(19)
Ch-M'-Ch Angle / °	111.0(1)	100.2(1)	101.0(1)	116.2(6)
M'-Ch bond length /°	2.536(2)	2.749(3)	2.760(2)	2.586(10)

Table S2. Selected bond distances and angles derived from Rietveld refinement to X-ray powder diffraction patterns. *M* = Sc, In or Y; *M`* = Cu or Ag; *Ch* = S or Se. Also included are the heights of the chalcogenide and oxide blocks. Errors are two sigma.

	E_{hull} (meV/atom)	a (= b) (Å)	c (Å)	α,β,γ (°)	Vol. (Å³)	M-Ch-M (°)	Ba-Ba (Å)	Ba-O (Å)	M-O (Å)	Decomposes to
$[Cu_2S_2][Ba_3Sc_2O_5]$*	0	4.12	26.92	90	457.76	117.49	7.94	2.68, 2.92, 3.06	1.98, 2.08	
$[Cu_2S_2][Ba_3In_2O_5]$*	0	4.18	27.19	90	476.06	119.62	8.13	2.69, 2.96, 3.16	2.08, 2.11	
$[Cu_2S_2][Ba_3Y_2O_5]$*	46.2	4.34	26.48	90	498.31	124.55	8.06	2.70, 3.07, 3.25	2.13, 2.19	BaY_2O_4, $BaSO_4$, BaS, Cu
$[Cu_2S_2][Ba_3La_2O_5]$*	131.6	4.49	27.18	90	547.9	130.68	8.61	2.73, 3.17	2.28, 2.32	BaO, $BaSO_4$, BaS, La_2O_3, Cu
$[Cu_2Se_2][Ba_3Sc_2O_5]$	0	4.16	27.49	90	476.5	112.99	7.88	2.69, 2.94, 3.06	1.97, 2.10	
$[Cu_2Se_2][Ba_3In_2O_5]$*	0	4.20	27.73	90	494.95	114.91	8.04	2.70, 2.99, 3.16	2.07, 2.13	
$[Cu_2Se_2][Ba_3Y_2O_5]$	0	4.36	27.20	90	518.14	119.12	8.03	2.71, 3.09, 3.25	2.12, 2.20	
$[Cu_2Se_2][Ba_3La_2O_5]$	76.3	4.52	27.80	90	567.94	124.8	8.55	2.75, 3.2	2.30, 2.31	$BaCu_2O_2$, BaSe, La_2O_3
$[Ag_2S_2][Ba_3Sc_2O_5]$	2.1	4.17	27.75	90	482.39	104.91	7.85	2.69, 2.95, 3.05	1.97, 2.10	$BaSc_2O_4$, $BaSO_4$, BaS, Ag
$[Ag_2S_2][Ba_3In_2O_5]$	8.5	4.23	27.96	90	499.82	106.96	8.03	2.70, 2.99, 3.16	2.08, 2.13	$Ba_2In_2O_5$, BaS, In_2O_3, $BaSO_4$, Ag
$[Ag_2S_2][Ba_3Y_2O_5]$	45.7	4.37	27.36	90	523.64	111.64	7.98	2.71, 3.09, 3.24	2.13, 2.20	BaY_2O_4, $BaSO_4$, BaS, Ag
$[Ag_2S_2][Ba_3La_2O_5]$	120.5	4.54	27.91	90	575.75	117.62	8.45	2.75, 3.21	2.30, 2.30	BaO, $BaSO_4$, BaS, La_2O_3, Ag
$[Ag_2Se_2][Ba_3Sc_2O_5]$	0	4.20	28.43	90	501.88	101.64	7.81	2.69, 2.97, 3.06	1.97, 2.12	
$[Ag_2Se_2][Ba_3In_2O_5]$	0	4.26	28.59	90	519.51	103.56	7.97	2.70, 3.01, 3.15	2.07, 2.15	
$[Ag_2Se_2][Ba_3Y_2O_5]$	0	4.40	28.16	90	544.42	107.65	7.98	2.72, 3.11, 3.24	2.12, 2.21	
$[Ag_2Se_2][Ba_3La_2O_5]$	54.7	4.56	28.71	90	596.77	112.77	8.47	2.77, 3.22	2.29, 2.31	BaO, $BaSeO_3$, BaSe, La_2O_3, Ag

Table S3 Energy above the convex hull of the compositional phase diagram, cell lattice parameters, atomic distances and angles calculated for each compound using the PBEsol functional. * Results for these compounds are in agreement with previously reported values and are recalculated and included here for completeness.

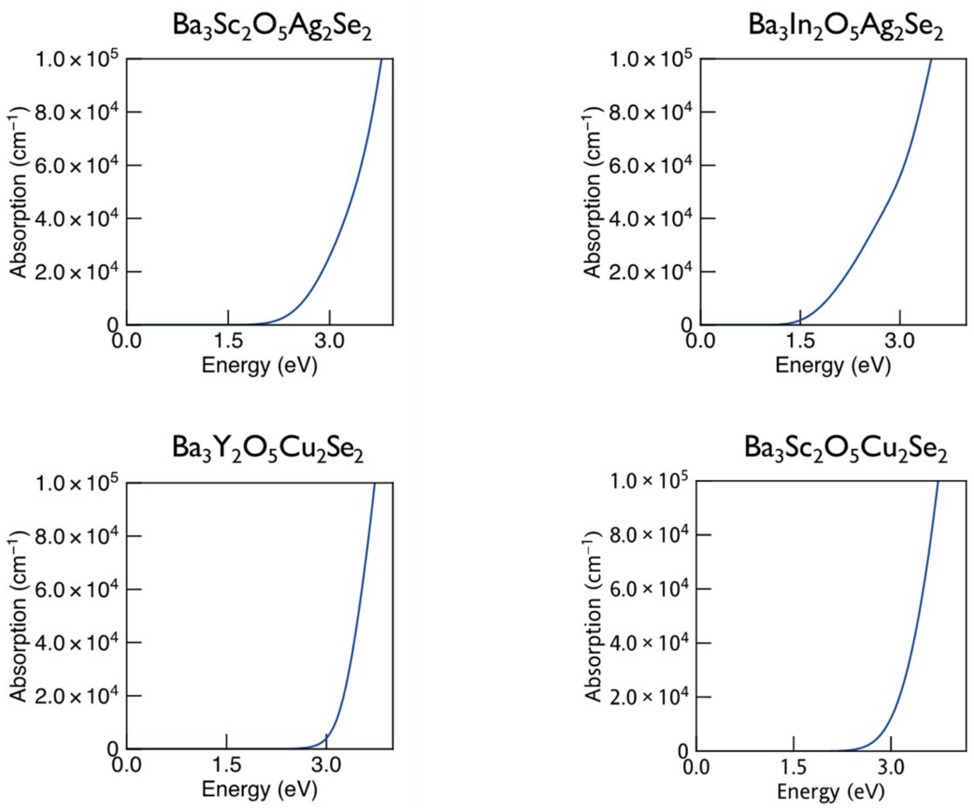

Figure S2. Optical absorption plots calculated calculated using the real and imaginary parts of the dielectric constant calculated through a Kramers-Kronig transformation and a summation over the unoccupied bands, respectively.

Attempted Composition	Chi^2	Actual composition					
$Ba_3Y_2O_5Cu_2S_2$	1.49	Y_2O_3 (22.4%)	BaS (31.7%)	Cu (17.0%)	$BaSO_4$ (11.4%)	$BaYO_4$ (17.5 %)	
$Ba_3La_2O_5Cu_2S_2$	1.14	La_2O_3 (33.6%)	BaS (28.2%)	Cu (21.8%)	$BaSO_4$ (9.7%)	$BaCO_3$ (6.6%)	
$Ba_3La_2O_5Cu_2Se_2$	1.69	La_2O_3 (34.9%)	BaSe (26.4%)	Cu (38.7%)			
$Ba_3La_2O_5Ag_2Se_2$	1.39	La_2O_3 (30.6%)	BaSe (55.9%)	Ag (11.9%)	$BaCO_3$ (3.3%)		
$Ba_3Sc_2O_5Ag_2S_2$	1.81	Sc_2O_3 (20.7%)	BaS (49.0%)	Ag (15.5%)	BaSO4 (14.7%)		
$Ba_3In_2O_5Ag_2S_2$	1.23	In_2O_3 (31.1%)	BaS (31.4%)	Ag (10.7%)	$BaSO_4$ (21.2%)	$Ba_2In_2O_5$ (5.5%)	
$Ba_3Y_2O_5Ag_2S_2$	1.21	Y_2O_3 (37.6%)	BaS (37.3%)	Ag (11.9%)	$BaSO_4$ (13.2%)		
$Ba_3La_2O_5Ag_2S_2$	1.2	La_2O_3 (38.0%)	BaS (38.8%)	Ag (13.5%)	$BaSO_4$ (9.6%)		

Table S4: Details of the refinement of the attempted compounds which could not be synthesized, and resultant composition, with Weight% of phases identified from Rietveld Refinement.

Formula	Γ–N / m_e	Γ–X / m_e
$Ba_3Y_2O_5Cu_2Se_2$	0.46	0.5
$Ba_3Sc_2O_5Cu_2Se_2$	N/A	0.45
$Ba_3Sc_2O_5Ag_2Se_2$	0.56	0.44
$Ba_3In_2O_5Ag_2Se_2$	0.56	0.37

Table S5. Light hole effective masses for the newly reported compounds, broken down by reciprocal space direction. Appropriate Brillouin diagram can found at *https://www.cryst.ehu.es/cgi-bin/cryst/programs/nph-kv-list?gnum=139&fig=f4ommmig&what=data*. *k-point coordinates in reciprocal space are:*
Γ: 0.0 0.0 0.0 ; Z: 0.5 0.5 -0.5 ; N: 0.0 0.5 0.0 ; P: 0.25 0.25 0.25 ; X: 0.0 0.0 0.5
